# Artificial Intelligence vs. Human Readers in Contrast-Enhanced Harmonic Imaging Endoscopic Ultrasound Interpretation of Solid Pancreatic Masses: A Multicenter Interobserver Study

**DOI:** 10.3390/jcm15093556

**Published:** 2026-05-06

**Authors:** Nicoleta Podină, Lucian Gheorghe Gruionu, Anca Udriștoiu, Elena Codruța Gheorghe, Voicu Rednic, Alina Liliana Constantin, Maria Simona Badiu, Cristian George Țieranu, Nona Bejinariu, Cristina Pojoga, Claudia Hagiu, Andrada Seicean, Adrian Săftoiu

**Affiliations:** 1 Department of Gastroenterology, “Carol Davila” University of Medicine and Pharmacy, 050474 Bucharest, Romania; nicoletapodinaa@gmail.com (N.P.); simona.badiu@yahoo.com (M.S.B.); adrian.saftoiu@umfcd.ro (A.S.); 2 Department of Gastroenterology, Ponderas Academic Hospital, 014142 Bucharest, Romania; 3 Faculty of Automation, Computers and Electronics, University of Craiova, 200585 Craiova, Romania; lgruionu@gmail.com; 4 Faculty of Mechanics, University of Craiova, 200585 Craiova, Romania; anca_soimu@yahoo.com; 5 Faculty of Medicine, University of Medicine and Pharmacy Craiova, 200349 Craiova, Romania; constantinescu.codruta@yahoo.com; 6 Department of Gastroenterology, Regional Institute of Gastroenterology and Hepatology, “Iuliu Hațieganu” University of Medicine and Pharmacy, 400162 Cluj-Napoca, Romania; rednicvoicu@gmail.com (V.R.); cristinapojoga@yahoo.com (C.P.); claudiahagiu@yahoo.com (C.H.); andradaseicean@gmail.com (A.S.); 7 Department of Gastroenterology and Hepatology, Elias Emergency University Hospital, 011461 Bucharest, Romania; 8 Santomar OncoDiagnostics, Regina Maria, 400000 Cluj-Napoca, Romania; nonarebei@yahoo.com; 9 Department of Clinical Psychology and Psychotherapy, International Institute for Advanced Study of Psychotherapy and Applied Mental Health, Babeș-Bolyai University, 400015 Cluj-Napoca, Romania

**Keywords:** artificial intelligence, contrast-enhanced harmonic endoscopic ultrasound, pancreatic cancer, diagnostic accuracy, interobserver variability, convolutional neural network, deep learning

## Abstract

**Background/Objectives:** Contrast-enhanced harmonic imaging endoscopic ultrasound (CHI-EUS) is a valuable tool for characterizing solid pancreatic tumors. However, interobserver variability remains a significant limitation in clinical interpretation. Artificial intelligence (AI) may offer objective, reproducible assessments, potentially enhancing diagnostic performance. This study compared the diagnostic accuracy and interobserver agreement of nine physicians with varying CHI-EUS experience levels vs. a dedicated AI system and a general-purpose large language model (ChatGPT) on the same 118 histologically confirmed cases. **Methods:** We conducted a prospective, multicenter, observer-blinded study involving 118 CHI-EUS video cases of histologically confirmed (EUS-FNB) focal pancreatic masses from three tertiary care centers in Romania. Nine readers were stratified into three groups: trainees (<5 years CHI-EUS experience), intermediates (5–10 years), and experts (>10 years). All readers and two AI models received standardized, anonymized 2 min CHI-EUS video clips. A dedicated AI system used a convolutional neural network (CNN) for lesion segmentation and time–intensity curve (TIC) extraction, followed by a feedforward neural network (FNN) for classification. ChatGPT was separately evaluated on the same videos. Diagnostic metrics (accuracy, sensitivity, specificity, positive predictive value [PPV], negative predictive value [NPV], and AUROC) were calculated. Interobserver agreement was assessed using Fleiss’ and Cohen’s kappa statistics. **Results:** The dedicated AI system achieved an overall accuracy of 95.8% (sensitivity 96.6%; specificity 94.1%) in diagnosing pancreatic adenocarcinoma. Expert readers had a mean accuracy of 78.8% (sensitivity 86%, specificity 61%, and AUROC 0.74), intermediates 80.8% (sensitivity 83%, specificity 75%, and AUROC 0.84), and trainees had a mean accuracy of 67.2% (sensitivity 70%, specificity 60%, and AUROC 0.67). For the most-likely-diagnosis parameter, interobserver agreement was similar between intermediates (Fleiss’ κ = 0.407) and experts (κ = 0.389), while trainees showed lower agreement (κ = 0.203). ChatGPT correctly classified only 14.1% of PDAC cases. **Conclusions:** A specialized AI model for CHI-EUS video analysis can achieve expert-level performance and reduce diagnostic variability across experience levels. Integration of dedicated AI systems into CHI-EUS interpretation may enhance accuracy and serve as a valuable decision support tool in clinical and training settings.

## 1. Introduction

Endoscopic ultrasonography (EUS) enables detailed evaluation of the entire pancreatic parenchyma and is widely recognized as the most sensitive imaging modality for the detection and characterization of solid pancreatic tumors [1]. These comprise a diverse group of pathologies, including pancreatic ductal adenocarcinoma (PDAC), pancreatic neuroendocrine tumors (pNETs), inflammatory masses, and focal autoimmune pancreatitis. However, establishing an accurate differential diagnosis for solid pancreatic masses remains complex [2].

PDAC is the most common solid pancreatic tumor, accounting for over 85% of cases. Globally, pancreatic cancer ranks as the seventh leading cause of cancer-related mortality [3]. Given PDAC’s poor prognosis and significant therapeutic implications, accurate differentiation from other solid pancreatic lesions is critical for effective clinical management. Moreover, the detection of pancreatic cancers smaller than 10 mm, which are considered to influence long-term prognosis, remains challenging. This underscores the clinical value of EUS in identifying and diagnosing small pancreatic lesions at an early stage [4].

Contrast-enhanced harmonic imaging EUS (CHI-EUS) plays an important role in improving the diagnostic accuracy of solid pancreatic tumor evaluation by providing real-time information on vascularity [5]. Several studies have reported that the combination of CHI-EUS and endoscopic ultrasound-guided fine-needle aspiration biopsy (EUS-FNAB) can further improve diagnostic accuracy for pancreatic cancer [6,7,8]. CHI-EUS supports the qualitative evaluation of pancreatic masses using second-generation ultrasound contrast agents, such as SonoVue^®^ or Sonazoid^®^. These agents react to low acoustic power by generating a second harmonic signal, which provides a contrast effect lasting for several minutes. On CHI-EUS, solid pancreatic masses typically exhibit one of four enhancement patterns: non-enhancement, hypo-enhancement, iso-enhancement, or hyper-enhancement. These patterns are useful for differentiation, with hypo-enhancement commonly seen in adenocarcinomas, iso-enhancement in inflammatory lesions, and hyper-enhancement in pNETs [9,10].

Despite its demonstrated value in assessing solid pancreatic tumors, it remains unclear how reproducible CHI-EUS findings are among different examiners. Some studies have reported interobserver agreement (IOA) in the evaluation of solid pancreatic masses by CHI-EUS, highlighting moderate consistency in lesion characterization but also revealing variability in interpreting contrast enhancement patterns among different observers [11,12,13,14].

The growing implementation of artificial intelligence (AI) in medical imaging has led to the development of advanced AI-based models capable of processing large-scale EUS datasets, demonstrating promising potential in improving diagnostic accuracy for pancreatic lesions [15,16].

Recent research has demonstrated that an AI-based diagnostic approach, combining convolutional neural networks (CNNs) for automated lesion detection and segmentation with feedforward neural networks (FNNs) trained on quantitative time–intensity curve (TIC) parameters from CHI-EUS, can reliably differentiate PDAC from other solid pancreatic masses. By integrating advanced image analysis with perfusion-based metrics such as peak intensity, wash-in and washout rates, and perfusion indices, this method achieved high diagnostic accuracy and shows considerable promise as a complementary tool to conventional EUS-based evaluation [17]. Therefore, AI may offer objective, reproducible assessments, potentially enhancing diagnostic performance.

This study assessed diagnostic accuracy and interobserver agreement among physicians with varying levels of CHI-EUS expertise and compared their performance both with a ChatGPT-based interpretation model and with a dedicated AI system specifically trained for CHI-EUS video analysis.

## 2. Materials and Methods

### 2.1. Study Population and Design

We conducted a prospective, multicenter, observer-blinded comparative study involving 118 CHI-EUS video cases of histologically confirmed focal pancreatic masses. CHI-EUS video recordings were collected from EUS procedures performed at three tertiary care centers in Romania (Elias Emergency University Hospital, Bucharest; Ponderas Academic Hospital, Bucharest; Regional Institute of Gastroenterology and Hepatology, Cluj-Napoca).

All 118 patients with solid pancreatic masses were assessed using CHI-EUS. The EUS examinations were carried out with a linear array Olympus EUS probe (GF-UCT 180; Olympus Medical Systems, Tokyo, Japan) attached to a Fujifilm ultrasound platform (Arietta 850; FUJIFILM Healthcare, Tokyo, Japan), which includes a contrast harmonic imaging module. Experienced endosonographers performed all procedures. SonoVue^®^ (sulfur hexafluoride microbubbles; Bracco Diagnostics Inc., Milan, Italy) was used as the contrast agent for CHI-EUS in every case.

The gold standard for diagnosis was histopathological confirmation obtained through EUS-guided fine-needle biopsy (EUS-FNB) in all 118 cases. All diagnoses were based on tissue histology; no cases relied solely on cytology or clinical follow-up alone. Of the 118 cases included, 85 (72.0%) were confirmed as PDAC, 13 (11.0%) were confirmed as neuroendocrine tumors, 12 (10.2%) were confirmed as chronic pancreatitis, and 8 (6.8%) were confirmed as other rare lesions (including solid pseudopapillary neoplasms, metastases, and lymphoma).

### 2.2. Reader Groups and Dataset Structure

A total of 9 endoscopists from the 3 participating centers were enrolled as readers and stratified into three groups based on their experience with EUS and CHI-EUS: Group A—trainees (less than 5 years of CHI-EUS experience; *n* = 3); Group B—intermediates (5–10 years; *n* = 3); and Group C—experts (more than 10 years; *n* = 3).

A standardized 2 min video sequence was recorded for each patient, starting immediately after SonoVue^®^ administration. Each video was assigned a random identification number. The readers were fully blinded to the patients’ clinical history, pathological diagnosis, and to each other’s assessments. Standardized, anonymized video clips (including arterial and venous phases) were presented in randomized order. Readers were given unlimited time to review the videos and were allowed to replay them without restriction. No clinical information was provided to the readers.

### 2.3. ChatGPT Agent Mode Evaluation

ChatGPT was evaluated as an exploratory general-purpose AI comparator using the ChatGPT Agent Mode (ChatGPT Agent Mode, July 2025 release) through the OpenAI web interface (prompted on 31 July 2025). The evaluation was performed on the same anonymized CHI-EUS video dataset used for human readers and the dedicated AI system. Agent Mode was used as a visual-agent workflow rather than a dedicated medical imaging model. No model training, fine-tuning, case-specific calibration, segmentation algorithm, time–intensity curve extraction, or quantitative perfusion analysis was performed for ChatGPT. Therefore, the results should be interpreted as a single-session exploratory assessment of ChatGPT Agent Mode as available on 31 July 2025, not as a locked benchmark of a specific API model version.

The input consisted of 118 anonymized CHI-EUS video clips, each assigned a study-specific case identifier. Each clip represented a standardized contrast-enhanced harmonic EUS sequence obtained after SonoVue^®^ administration and included the arterial and venous phases. No clinical history, laboratory data, histopathological diagnosis, previous reader interpretation, or outcome information was provided to ChatGPT during video interpretation. The video files were made available to ChatGPT Agent Mode in a single working environment, and the agent was instructed to review the cases sequentially and complete one structured row per video in a predefined spreadsheet template.

We used a standardized prompt to analyze the movies and populate the final database template as follows:

“Review each anonymized CHI-EUS movie sequentially. For every case, inspect the full video sequence and complete one row in the spreadsheet. Use only the provided Case ID and do not use any clinical or histopathological information. For each video, report:

1. CEH-EUS Image/Movie Review—Arterial Phase
  - Lesion enhancement pattern (hyperenhancing homogeneous/heterogeneous, rim enhancement, isoenhancing, hypoenhancing, no enhancement);
  - Washout onset (early <30 s, intermediate 30–60 s, late >60 s, no washout);
  - Arterial-phase pattern interpretation (pancreatic adenocarcinoma, neuroendocrine tumor, autoimmune pancreatitis, cystic neoplasm, metastatic lesion, indeterminate).
2. CEH-EUS Image/Movie Review—Venous Phase
  - Lesion enhancement relative to the surrounding pancreatic tissue (hyperenhancing, isoenhancing, hypoenhancing, no enhancement);
  - Washout characteristics (progressive washout, punctate washout, rim washout, no washout);
  - Venous-phase pattern interpretation (pancreatic adenocarcinoma, neuroendocrine tumor, autoimmune pancreatitis, cystic neoplasm, metastatic lesion, indeterminate).
3. Differential Diagnosis—Final Interpretation
  - Most likely diagnosis selected from several categories (pancreatic ductal adenocarcinoma, pancreatic neuroendocrine tumor, chronic pseudotumoral pancreatitis/autoimmune pancreatitis, pancreatic metastatic tumor, or cystadenocarcinoma);
  - Confidence level (high ≥90% sure, moderate 60–90% sure, low <60% sure);
  - Time taken for full interpretation (auto-recorded for AI);
  - Brief free-text comment explaining the interpretation.

If uncertain, select the closest diagnostic category and lower the confidence level. Do not create additional diagnostic categories.”

For each case, ChatGPT Agent Mode visually inspected the video, generated structured descriptors of arterial enhancement, lesion-to-parenchyma enhancement, washout behavior, and venous-phase appearance, and entered the output directly into the spreadsheet. One final diagnosis was recorded per case. No repeated prompting, majority voting, ensemble interpretation, or post hoc correction of the diagnostic label was performed before comparison with the reference diagnosis.

For statistical analysis, ChatGPT’s categorical diagnosis was mapped to a binary endpoint of PDAC vs. non-PDAC. Minor capitalization and spelling variants of the same non-PDAC diagnostic category were normalized before analysis. The free-text AI explanation field was not used for diagnostic scoring.

Because ChatGPT Agent Mode is a commercial web-based agentic system, THE backend model and tool orchestration of which may change over time, the exact backend model/version could not be retrospectively recovered from the retained files. The analysis should, therefore, be interpreted as a single-run exploratory assessment of a general-purpose visual-agent workflow, not as a reproducible benchmark of a locked medical imaging model.

### 2.4. Dedicated AI System: Architecture, Training, and Inference Workflow

The AI system had been developed before the present reader-comparison analysis, and its architecture and diagnostic threshold were fixed before evaluation against human observers [17,18]. Thus, the current 118-case dataset was used as an independent external validation set obtained from three centers in Romania; this was completely different than the development set used before, which was generated entirely from Orlando Health Digestive Health Institute. The present 118-case dataset represented the final eligible subset of the previously described 120-case multicenter CEH-EUS dataset after exclusion of technically inadequate or incomplete cases. Therefore, the AI results should be interpreted as performance on a curated multicenter test external validation cohort.

The dedicated AI system was developed as a computer-assisted diagnostic framework for the characterization of solid pancreatic masses on contrast-enhanced harmonic EUS. The model used a two-stage workflow combining automated image segmentation with quantitative contrast-enhancement analysis.

In the first stage, a convolutional neural network-based detection and segmentation model was trained on labeled non-contrast EUS frames to identify the pancreas and tumor regions. During development, CNN-based object detection/segmentation architectures, including R-CNN-type and YOLO-type approaches, were evaluated for lesion localization. The final trained detector was applied to new EUS video cases to identify regions of interest corresponding to the pancreatic parenchyma and the solid tumor on the non-contrast component of the EUS image.

The detected tumor ROI was then mapped onto the corresponding contrast-enhanced harmonic EUS sequence. Time–intensity curve analysis was performed within the mapped ROI to quantify contrast-enhancement dynamics over time. Extracted TIC parameters included temporal metrics, such as time to peak and rise time; intensity-based metrics, such as peak intensity and wash-in intensity; slope-derived metrics, including wash-in and washout rates; area-based parameters, including area under the enhancement curve; and perfusion-related variables, including perfusion index and blood-flow-related parameters. These parameters were selected to capture both arterial enhancement and venous washout behavior of the lesion.

In the second stage, the extracted TIC parameters were used as input variables for a feedforward neural network classifier. The FNN generated a probability-based classification for pancreatic ductal adenocarcinoma vs. non-adenocarcinoma solid pancreatic lesions. For the present analysis, the AI output was dichotomized as PDAC or non-PDAC using a decision threshold fixed before evaluation of the reader–study dataset. The threshold was not modified after comparison with human reader performance.

The final integrated model, therefore, consisted of: automated pancreas and tumor detection on EUS frames; mapping of the detected ROI to the contrast-enhanced sequence; TIC extraction from the lesion ROI; and FNN-based diagnostic classification. No manual correction of AI-generated ROIs or diagnostic outputs was performed during testing.

Video quality was assessed before inclusion in the final analysis. Videos were considered inadequate if the lesion was not continuously visible, if the contrast-enhancement sequence was incomplete, if arterial or venous phase information was missing, or if major motion or technical artefacts prevented reliable ROI mapping and TIC extraction. Such videos were excluded before the final analysis. In the final dataset, only videos fulfilling these predefined technical requirements were submitted to the AI pipeline.

### 2.5. Interpretation Protocol

Endoscopic ultrasonographers were instructed to analyze seven predefined parameters for each video clip. The interpretation of contrast enhancement timing followed the most recent European guidelines [19], which distinguish two primary phases commonly observed across organs: an arterial phase, occurring approximately 10–20 s after contrast administration and lasting up to 35–40 s, during which enhancement progressively increases, and a venous phase, beginning at around 30–45 s post-injection, characterized by an initial plateau followed by a gradual decline in intensity. SonoVue^®^ uptake, representing the overall enhancement of the lesion, was assessed in relation to the surrounding tissue and categorized into five patterns: homogeneous hyper-enhancement, heterogeneous hyper-enhancement, iso-enhancement, hypo-enhancement, and no enhancement. Furthermore, the washout dynamics, reflecting the rate at which SonoVue^®^ exited the lesion, were evaluated and grouped into four types: progressive, punctate, rim, and no washout.

### 2.6. Statistical Analysis

Diagnostic performance was evaluated for each reader group, the dedicated AI system, and ChatGPT using the following metrics: sensitivity, specificity, positive predictive value (PPV), negative predictive value (NPV), and area under the receiver operating characteristic curve (AUROC). The AUROC was computed from an ordinal probability-of-PDAC score constructed by combining each observer’s binary diagnostic classification with their three-level confidence rating, thereby enabling a graded assessment of diagnostic certainty. Interobserver agreement was assessed using Fleiss’ kappa (κ) for multi-reader agreement within and across groups, and Cohen’s kappa was assessed for pairwise comparisons. Interpretation of κ values followed the Landis and Koch classification [20]: slight (0.00–0.19), fair (0.20–0.39), moderate (0.40–0.59), substantial (0.60–0.79), or almost perfect (>0.80). A *p*-value < 0.05 was considered statistically significant. All kappa calculations and statistical analyses were performed using Python-based statistical tools (https://www.python.org/). As this was a prospective observational study with consecutive patient enrolment, a formal a priori sample size calculation was not performed. However, the sample size of 118 cases was considered adequate for the primary diagnostic comparison.

## 3. Results

### 3.1. Patient Characteristics

A total of 118 patients were included in this study. The diagnosis was based on an EUS-FNB histopathological examination in all cases. Of the 118 cases, 85 (72.0%) were confirmed as PDAC, 13 (11.0%) were confirmed as neuroendocrine tumors, 12 (10.2%) were confirmed as chronic pancreatitis, and 8 (6.8%) were confirmed as other rare lesions (including solid pseudopapillary neoplasms, metastases, and lymphoma).

### 3.2. Interobserver Agreement (IOA)

Interobserver agreement, as measured by Fleiss’ κ, varied across the five evaluated imaging parameters and the three reader groups, with overall values ranging from slight to fair agreement (Table 1). Agreement was highest for the most likely diagnosis and for the arterial phase pattern, and lowest for washout characteristics.

For the lesion enhancement pattern (lesion enhancement compared with surrounding parenchyma), overall agreement was fair (κ = 0.311), and was the highest among intermediate readers (κ = 0.422, moderate) and comparable between trainees (κ = 0.272) and experts (κ = 0.267), both classified as fair.

Washout characteristics demonstrated only slight overall agreement (κ = 0.036), with low κ values in every stratum. The expert group registered a negative Fleiss’ κ (κ = −0.217), reflecting systematic categorical divergence among senior readers rather than random disagreement; inspection of the marginal category distributions indicates that one expert predominantly labeled cases as “no washout” while the other two predominantly labeled them as “progressive washout”, pointing to heterogeneous operational definitions of washout among senior readers.

Both arterial-phase and venous-phase parameters demonstrated fair overall agreement (κ = 0.382 and κ = 0.298, respectively). Experts and intermediate readers reached comparable agreement on the arterial phase (κ = 0.392 and 0.396, both fair), while trainees were limited to slight concordance (κ = 0.188). For the venous phase, Experts showed the strongest concordance (κ = 0.404, moderate), followed by intermediate readers (κ = 0.335, fair) and trainees (κ = 0.177, slight).

Agreement for the most likely diagnosis was fair overall (κ = 0.322). Moderate agreement was observed in the intermediate group (κ = 0.407), while experts and trainees remained within the fair range, at the upper (κ = 0.389) and lower (κ = 0.203) ends, respectively, suggesting an experience-dependent gradient in agreement.

Most κ values were statistically significant at *p* < 0.01, indicating that observed agreements were unlikely to have arisen by chance. The exception was washout characteristics, where the trainee (*p* = 0.83) and overall (*p* = 0.29) estimates were not statistically distinguishable from zero, consistent with the absence of structured agreement on this parameter across reader groups (Table 1).

### 3.3. Diagnostic Performance of Human Observers

The diagnostic performance data for PDAC detection across the three physician experience strata are summarized in Table 2. Overall sensitivity was 79.7% and specificity was 65.0%, with a positive predictive value (PPV) of 85.4% and a negative predictive value (NPV) of 55.5%; the pooled area under the ROC curve (AUROC) across all nine observers was 0.754.

The intermediate group achieved the highest AUROC (0.845, 95% CI 0.797–0.884), followed by experts (0.737, 95% CI 0.674–0.791) and trainees (0.668, 95% CI 0.605–0.726). The 95% CIs of the intermediate and expert pooled AUROCs did not overlap, indicating a statistically discernible difference in discriminative ability between these two groups, with both substantially exceeding those of trainees.

Experts reached the highest sensitivity (85.9%), while intermediate readers showed the highest specificity (74.7%) and delivered a more balanced performance between sensitivity (83.1%) and specificity (74.7%), which accounts for their superior overall discriminative ability. Expert readers, by contrast, combined high sensitivity (85.9%) with a lower specificity (60.6%), reflecting a more inclusive pattern of PDAC calls.

Trainees demonstrated the lowest performance across all five metrics, particularly in terms of NPV (43.7%) and AUROC (0.668), compared with 63.2% and 62.5% NPV for intermediate and expert readers, respectively. Overall diagnostic accuracy followed the same ordering—intermediate: 80.8%; expert: 78.8%; trainee: 67.2%—underlining the effect of clinical experience on the ability to discriminate malignant from non-malignant lesions using CE-EUS.

### 3.4. ChatGPT Versus Human Observers

ChatGPT (Agent Mode) was prompted to review the imaging videos of all 118 included cases and to provide its most likely diagnosis using the same structured template as the physician observers. Its classifications were then compared against the final histology-confirmed reference diagnosis and those of the three human observer groups.

Of the 85 true PDAC cases, ChatGPT correctly identified only 12 (14.1%) and missed 73 (85.9%). Of the 33 non-PDAC cases, the model correctly classified 26 (78.8%), while 7 non-PDAC lesions were incorrectly labeled as PDAC (false positives). These results reflect the model’s very low sensitivity (14.1%, 95% CI 8.3–23.1) and a superficially acceptable specificity (78.8%, 95% CI 62.2–89.3) which, however, was driven almost entirely by a pervasive reluctance to call PDAC rather than by genuine discriminative ability—ChatGPT returned a PDAC diagnosis in only 19 out of 118 cases (16.1%), which is well below the sample prevalence of 72.0%. The resulting PPV (63.2%) and NPV (26.3%) were both clinically uninformative (Table 3). The pooled AUROC was 0.503 (95% CI 0.378–0.628), an interval that crosses 0.5 and is, therefore, statistically indistinguishable from chance.

When compared with the human observer groups (Table 4), the overall diagnostic accuracy for PDAC was substantially higher for all three physician strata than for ChatGPT: intermediate readers achieved the highest accuracy (80.8%), followed by experts (78.8%) and trainees (67.2%), while ChatGPT correctly classified approximately one-third of cases (32.2%). The pooled AUROC for human observers (0.754, 95% CI 0.720–0.785) was markedly superior to that of ChatGPT (0.503, 95% CI 0.378–0.628), and the 95% CIs of the two estimates did not overlap. Even the least-experienced human group (Trainees, AUROC 0.668) exceeded ChatGPT’s AUROC, highlighting a considerable gap between the current general-purpose AI model and human readers for this CE-EUS interpretation task.

### 3.5. Diagnostic Performance of the Dedicated AI-Based CHI-EUS System

In addition to the evaluation of human observers and ChatGPT, a dedicated AI system based on convolutional and feedforward neural networks was tested on the same dataset of 118 anonymized CHI-EUS videos obtained from patients with solid pancreatic masses across three tertiary referral centers in Romania.

The AI system used a CNN for pancreas and tumor segmentation, followed by TIC extraction, which served as input for an FNN classifier. When evaluated on the 118-case external test dataset, entirely independent from the training data, the AI system demonstrated superior diagnostic performance compared with both human observers and ChatGPT. The system achieved a sensitivity of 96.6%, specificity of 94.1%, and an overall accuracy of 95.8% in detecting pancreatic adenocarcinoma.

These findings support the strong potential of AI-based CHI-EUS analysis as an adjunct to human interpretation, enhancing diagnostic consistency and accuracy in clinical practice.

## 4. Discussion

This study provides an integrated assessment of diagnostic performance in CHI-EUS by comparing three groups of human observers with different levels of experience, a general-purpose language model (ChatGPT), and a dedicated AI-based system, all applied to the same 118 histologically confirmed cases. The findings reveal important insights regarding the current state of CHI-EUS interpretation and the potential role of artificial intelligence in enhancing diagnostic accuracy and consistency.

### 4.1. Human Observer Performance in the Context of Published Meta-Analyses

The overall human observer performance in our study (80% sensitivity; 65% specificity) requires careful contextualization within the broader literature on CHI-EUS diagnostic accuracy. At first glance, these values may appear modest compared to the pooled estimates reported in recent meta-analyses. Yamashita et al. reported a pooled sensitivity and specificity of 93% and 80%, respectively, for CHI-EUS with enhancement pattern analysis in pancreatic cancer diagnosis [21]. Similarly, Li et al. found a pooled sensitivity of 92% and specificity of 80% in their systematic review [22]. Gong et al. reported comparable results with a pooled sensitivity of 94% and specificity of 82% for contrast-enhanced EUS in differentiating pancreatic masses [23].

However, these meta-analytic estimates reflect highly selected study populations, often with retrospective designs, expert-only interpretation, and publication bias favoring positive results. In contrast, our study deliberately included readers across the full spectrum of experience levels, from trainees with less than 5 years of CHI-EUS experience to experts with more than 10 years of experience, thus employing a prospective, multicenter design with real-world case complexity. This methodological approach provides a more realistic assessment of CHI-EUS performance as it would be encountered in routine clinical practice, where not all interpretations are performed by highly experienced specialists.

Importantly, when examining our expert readers in isolation, their performance (86% sensitivity, 61% specificity, and 78.8% overall accuracy) approaches the pooled estimates from meta-analyses, particularly in terms of sensitivity. The lower specificity observed in our expert group may reflect the inherent challenge of distinguishing inflammatory masses and well-differentiated neuroendocrine tumors from adenocarcinoma, a diagnostic dilemma that is often underrepresented in highly curated meta-analytic datasets. Furthermore, meta-analyses by D’Onofrio et al. [24] and Mei et al. [25] reported similar patterns, reinforcing that CHI-EUS specificity is inherently lower in heterogeneous real-world populations.

### 4.2. Interobserver Variability and Clinical Implications

Interobserver variability remained a major limitation of CHI-EUS interpretation in our study, with fair overall agreement for lesion enhancement pattern, arterial-phase and venous-phase patterns, and the most likely diagnosis, and only slight overall agreement for washout characteristics (Table 1). This finding is consistent with previous reports in the literature [11,12,13,14] and highlights the subjective nature of qualitative CHI-EUS interpretation. For the most-likely-diagnosis parameter, agreement was highest among intermediates (Fleiss’ κ = 0.407, moderate), followed closely by experts (κ = 0.389, fair) and trainees (κ = 0.203, fair); no reader group reached substantial agreement. These results emphasize that CHI-EUS interpretation is a skill that improves significantly with experience and training, but even among experts, perfect concordance is not achieved.

The clinical implications of this variability are significant. In centers where CHI-EUS interpretation is performed by less experienced operators, diagnostic accuracy may be compromised, potentially leading to delayed diagnosis or unnecessary interventions. This underscores the need for standardized training protocols, quality assurance programs, and the potential integration of objective decision support tools to reduce reader-dependent variability.

### 4.3. Comparison with CNN-Based AI Systems

The dedicated AI system in our study achieved remarkable performance with 95.8% overall accuracy, 96.6% sensitivity, and 94.1% specificity, substantially outperforming all human reader groups. This performance is consistent with other recently published CNN-based systems specifically designed for CHI-EUS analysis of pancreatic masses.

Tang et al. developed the CHI-EUS MASTER system, a deep learning-based model utilizing a ResNet-50 backbone architecture for automated pancreatic mass diagnosis [26]. Their system achieved 93.5% accuracy in distinguishing PDAC from other pancreatic lesions, with a sensitivity of 94.2% and a specificity of 91.8%. The slightly higher accuracy achieved by our AI system (95.8% vs. 93.5%) may reflect differences in training dataset composition, architectural refinements, or the specific preprocessing and feature extraction strategies employed.

Yi et al. developed an interpretable deep learning model specifically for distinguishing pancreatic neuroendocrine tumors from pancreatic cancer using standard EUS images [27]. Their model achieved an AUC of 0.948 in the training cohort and 0.795 in the test cohort. While their study focused on standard EUS rather than CHI-EUS, their work demonstrates the broader applicability of deep learning approaches to pancreatic mass characterization. The authors emphasized the importance of model interpretability using Gradient-Weighted Class Activation Mapping (Grad-CAM) and Shapley Additive Explanations (SHAP) techniques that enhance clinical trust and facilitate understanding of model decision-making processes.

The convergence of results across these independent studies, including our own, provides strong evidence that CNN-based AI systems can achieve expert-level or super-expert-level performance in CHI-EUS interpretation. Several important considerations need to be addressed before widespread clinical adoption. First, all studies were conducted in controlled research settings with carefully curated datasets. Thus, real-world implementation will require validation across diverse patient populations, imaging equipment, and clinical workflows. Second, integration of AI systems into clinical practice must be accompanied by appropriate regulatory oversight, quality assurance mechanisms, and ongoing performance monitoring. Third, AI should augment rather than replace human expertise, particularly in complex or ambiguous cases where clinical context and multimodal integration are essential.

### 4.4. ChatGPT Performance and Limitations of General-Purpose Language Models

ChatGPT performed considerably worse than all human observers, correctly identifying only 14.1% of PDAC cases. Although capable of generating structured textual interpretations and recognizing simple enhancement descriptors, the model lacked the ability to analyze CHI-EUS perfusion dynamics or integrate subtle contextual cues that are essential for reliable pancreatic tumor characterization. This underscores the fundamental difference between general-purpose language models and dedicated imaging-trained AI systems.

General-purpose language models like ChatGPT are trained primarily on text data and lack the specialized visual processing capabilities required for medical image interpretation. While these models can provide useful support for tasks such as report generation, literature summarization, and patient education, they are not suitable for primary diagnostic interpretation of complex imaging modalities like CHI-EUS. The poor performance of ChatGPT in our study serves as an important cautionary note against the indiscriminate application of general AI tools to specialized medical imaging tasks.

### 4.5. Clinical Integration and Future Directions

The findings of this study have several important implications for clinical practice and future research. First, the substantial performance gap between experienced and trainee readers highlights the need for structured training programs in CHI-EUS interpretation. Simulation-based training, case-based learning modules, and mentored interpretation sessions may help accelerate skill acquisition and reduce interobserver variability.

Second, the superior performance of dedicated AI systems suggests that AI-assisted interpretation could serve as a valuable decision support tool, particularly in centers with limited access to expert CHI-EUS readers. Potential clinical use cases include: (a) real-time decision support during EUS procedures, providing automated enhancement pattern analysis and preliminary diagnostic suggestions; (b) a training and educational tool for less experienced endosonographers, offering immediate feedback and comparison with expert-level interpretation; (c) a second-reader or quality assurance system to flag discordant interpretations for expert review; (d) a triage system for identifying high-uncertainty cases requiring additional diagnostic workup; and (e) integration into structured EUS reporting platforms to standardize diagnostic documentation.

Third, the development of interpretable AI models using techniques such as Grad-CAM and SHAP is essential for building clinical trust and facilitating regulatory approval. Clinicians need to understand not only what an AI system predicts, but also why it makes specific predictions, enabling them to critically evaluate AI recommendations and identify potential failure modes.

### 4.6. Limitations

This study has several limitations that should be acknowledged. First, although data were collected prospectively, all examinations were performed using a single EUS platform (Olympus GF-UCT 180 with Fujifilm Arietta 850), which may limit the generalizability of findings to settings using different equipment manufacturers or probe configurations. Multi-platform validation is necessary before broader clinical adoption. Second, this study included patients from three Romanian centers, potentially limiting external validity across different populations, disease prevalences, and healthcare settings. Third, no formal a priori sample size calculation was performed. Thus, the sample of 118 cases, while adequate for the primary diagnostic comparison, may have limited the detection of smaller effect sizes in subgroup analyses. Fourth, the use of a single contrast agent (SonoVue^®^) precludes conclusions regarding AI performance with other agents such as Sonazoid^®^. Fifth, future studies should incorporate 95% confidence intervals for all diagnostic metrics and formal statistical tests (e.g., the McNemar test and DeLong test for ROC comparison) for pairwise performance comparisons. Finally, the potential impact of AI on clinical decision-making and patient outcomes remains to be evaluated in prospective interventional studies.

A further limitation concerns the validation level of the dedicated AI system. Although the AI workflow was locked before comparison with human readers, the relationship between the development cohort and the present reader–study cohort should be considered when interpreting the results. The present dataset represents a subset of the previously described multicenter AI external validation cohort obtained in three different centers; hence, the results should be regarded as a fully independent external validation. Nevertheless, larger external datasets acquired across different EUS platforms, contrast settings, operators, and institutions will be required before general clinical deployment.

## 5. Conclusions

This multicenter study shows that human observers with varying experience levels can achieve clinically meaningful diagnostic performance in CHI-EUS assessments of solid pancreatic masses, with an overall sensitivity of 80% and specificity of 65%. Diagnostic accuracy was similar between expert (78.8%) and intermediate readers (80.8%), whereas trainees demonstrated lower performance (67.2%), highlighting the impact that experience and training level have. Interobserver variability remained a significant limitation, with agreement ranging from slight to fair among readers, underscoring the subjective nature of qualitative CHI-EUS interpretation and the need for standardized training and quality assurance.

The dedicated AI system incorporating CNN segmentation and TIC-based quantitative perfusion analysis demonstrated superior performance, achieving 95.8% accuracy (96.6% sensitivity; 94.1% specificity). This performance is consistent with other recently published CNN-based systems, suggesting that AI-assisted CHI-EUS interpretation may achieve high diagnostic performance under controlled conditions. However, as this study evaluates standalone AI and human observer performance, it does not directly assess the impact of AI assistance on interobserver variability or clinical decision-making.

ChatGPT showed poor diagnostic performance in this task, correctly identifying only 14.1% of PDAC cases and achieving an overall binary classification accuracy of 32.2%, supporting the conclusion that general-purpose visual-agent tools should not be used for standalone CHI-EUS diagnosis. Continued development, multicenter validation, and regulatory evaluation of imaging-trained AI tools are essential before their integration into clinical practice can be considered.

## Figures and Tables

**Table 1:** Interobserver agreement for selected parameters by experience group.

Parameter	Group	Fleiss’ κ (95% CI)	Interpretation	*p*-Value
Lesion enhancement pattern	Trainee	0.272 (0.154–0.374)	Fair	<0.001
	Intermediate	0.422 (0.307–0.529)	Moderate	<0.001
	Expert	0.267 (0.155–0.375)	Fair	0.001
	Overall	0.311 (0.242–0.374)	Fair	<0.001
Washout characteristics	Trainee	0.018 (−0.051–0.090)	Slight	0.833
	Intermediate	0.141 (0.054–0.228)	Slight	0.046
	Expert	−0.217 (−0.278–−0.159)	Poor	<0.001
	Overall	0.036 (0.004–0.067)	Slight	0.296
Arterial phase pattern	Trainee	0.188 (0.095–0.271)	Slight	<0.001
	Intermediate	0.396 (0.218–0.548)	Fair	<0.001
	Expert	0.392 (0.274–0.494)	Fair	<0.001
	Overall	0.382 (0.263–0.485)	Fair	<0.001
Venous phase pattern	Trainee	0.177 (0.080–0.264)	Slight	0.001
	Intermediate	0.335 (0.241–0.419)	Fair	<0.001
	Expert	0.404 (0.282–0.510)	Moderate	<0.001
	Overall	0.298 (0.233–0.351)	Fair	<0.001
Most likely diagnosis	Trainee	0.203 (0.107–0.287)	Fair	<0.001
	Intermediate	0.407 (0.310–0.498)	Moderate	<0.001
	Expert	0.389 (0.268–0.496)	Fair	<0.001
	Overall	0.322 (0.251–0.381)	Fair	<0.001

**Table 2:** Diagnostic performance per observer group for PDAC detection.

Group	Sensitivity (95% CI)	Specificity (95% CI)	PPV (95% CI)	NPV (95% CI)	AUROC (95% CI)
Trainee	70.2 (64.3–75.5)	59.6 (49.7–68.7)	81.7 (76.1–86.3)	43.7 (35.6–52.1)	0.668 (0.605–0.726)
Intermediate	83.1 (78.1–87.2)	74.7 (65.4–82.3)	89.5 (84.9–92.8)	63.2 (54.2–71.4)	0.845 (0.797–0.884)
Expert	85.9 (81.1–89.6)	60.6 (50.8–69.7)	84.9 (80.0–88.7)	62.5 (52.5–71.5)	0.737 (0.674–0.791)
Overall	79.7 (76.7–82.4)	65.0 (59.4–70.2)	85.4 (82.7–87.8)	55.5 (50.2–60.6)	0.754 (0.720–0.785)

**Table 3:** Diagnostic performance of ChatGPT for PDAC detection.

Observer	Sensitivity (95% CI)	Specificity (95% CI)	PPV (95% CI)	NPV (95% CI)	AUROC (95% CI)
ChatGPT (Agent Mode)	14.1 (8.3–23.1)	78.8 (62.2–89.3)	63.2 (41.0–80.9)	26.3 (18.6–35.7)	0.503 (0.378–0.628)

**Table 4:** Head-to-head comparison of diagnostic performance: human observers versus ChatGPT (Agent Mode).

Observer	Sensitivity (95% CI)	Specificity (95% CI)	PPV (95% CI)	NPV (95% CI)	AUROC (95% CI)
Human observers	79.7 (76.7–82.4)	65.0 (59.4–70.2)	85.4 (82.7–87.8)	55.5 (50.2–60.6)	0.754 (0.720–0.785)
ChatGPT (Agent Mode)	14.1 (8.3–23.1)	78.8 (62.2–89.3)	63.2 (41.0–80.9)	26.3 (18.6–35.7)	0.503 (0.378–0.628)

## Data Availability

The data supporting the findings of this study are available from the corresponding author upon reasonable request.
